# Case Report: Response to *ALK*-TKIs in a metastatic lung cancer patient with morphological heterogeneity and consistent molecular features

**DOI:** 10.3389/fonc.2023.1209799

**Published:** 2023-08-11

**Authors:** Yu Yang, Han Liu, Tao-hua Liu, Xi-run Zheng, Bin Wu, Dong-jing Zhou, Guang-juan Zheng, Xiao-shu Chai

**Affiliations:** ^1^ Department of Pathology, The Second Clinical College of Guangzhou University of Chinese Medicine, Guangzhou, China; ^2^ Department of Image, The Second Clinical College of Guangzhou University of Chinese Medicine, Guangzhou, China; ^3^ Department of Oncology, The Second Clinical College of Guangzhou University of Chinese Medicine, Guangzhou, China

**Keywords:** adenosquamous carcinoma, metastasis lung cancer, histomorphology, *ALK* rearrangement, targeted therapy

## Abstract

Lung adenosquamous carcinoma (ASC) is a rare heterogeneous tumor containing two distinct components of adenocarcinoma (ADC) and squamous cell carcinoma (SQCC). The limited biopsy sampling of the primary tumor might have overlooked either the ADC component or the SQCC component, resulting in a misdiagnosis of pure histology. Genotyping for driver mutations is now routinely performed in clinical settings to identify actionable oncogenic mutations and gene arrangements. Additionally, somatic mutations can potentially serve as a marker of clonal relationships. We report a rare case of ASC lung cancer, in which metastases were identified as ADC, while the primary was initially diagnosed as SQCC based on a fibrobronchoscope brush biopsy. The primary and metastatic tumors shared *ALK* rearrangement and other mutations support they were derived from a single clone origin. Our hypothesis is that the primary tumor contained a minor component of ADC that was not present in the histologic sections of lung biopsy. After sequential *ALK*-tyrosine kinase inhibitor (TKI) targeted therapy, both the patient’s primary lung tumor and the site of metastatic subcutaneous nodules decreased in size, with the metastatic sites demonstrating more noticeable shrinkage. However, after 11 months of targeted therapy, the patient was found to be resistant to *ALK*-TKIs. Subsequently, the patient’s respiratory status deteriorated rapidly, and a cycle of immunotherapy and chemotherapy did not show efficacy. To the best of our knowledge, this is a very rare case of lung ASC, disseminated metastasizing, with distinct morphology between the primary and metastases. Different therapeutic effects of *ALK*-TKIs were observed in two different morphological sites, with the metastatic cutaneous lesions shrinking more significantly than the primary lung lesions, though they both harbor the same *EML4*-*ALK* rearrangement. This case may provide diagnostic and therapeutic insights into lung ASC.

## Introduction

It is widely believed that the morphology of metastases mirrors that of the primary site. However, the impression could be challenged. Discrepancies in morphology between the metastatic sites and the primary tumor would lead to misdiagnosis and further result in inappropriate therapy, ultimately leading to a worse prognosis. Non-small cell lung cancer (NSCLC) is a highly heterogeneous tumor and lung adenosquamous carcinoma (ASC) is an uncommon heterogeneous tumor containing two distinct components of adenocarcinoma (ADC) and squamous cell carcinoma (SQCC) (1). It is quite challenging to diagnose ASC due to the limitations of biopsy specimens or the predominance of a single histology. To our knowledge, there are very few publications reporting cases where the primary and metastases of lung cancer have different histologic features (2, 3).

Genotyping for driver mutations is now routinely performed in clinical settings to identify actionable oncogenic mutations and gene arrangements. The somatic mutations can also be employed as markers of clonal relationship to address the clinical challenge of distinguishing independent primary tumors from metastases in NSCLC (4, 5).

## Case presentation

**Figure 1:**
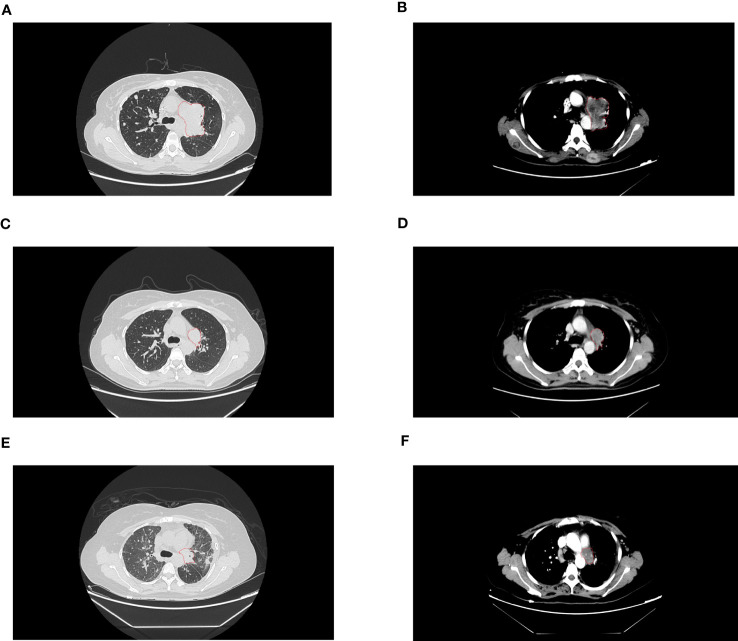
The lesions on computed tomography. **(A, B)** were before treatment of aletinib. **(C, D)** were partial response after 8 weeks treatment of aletinib. **(E, F)** were progressive disease after 11 months treatment of *ALK*-TKI.

**Figure 2:**
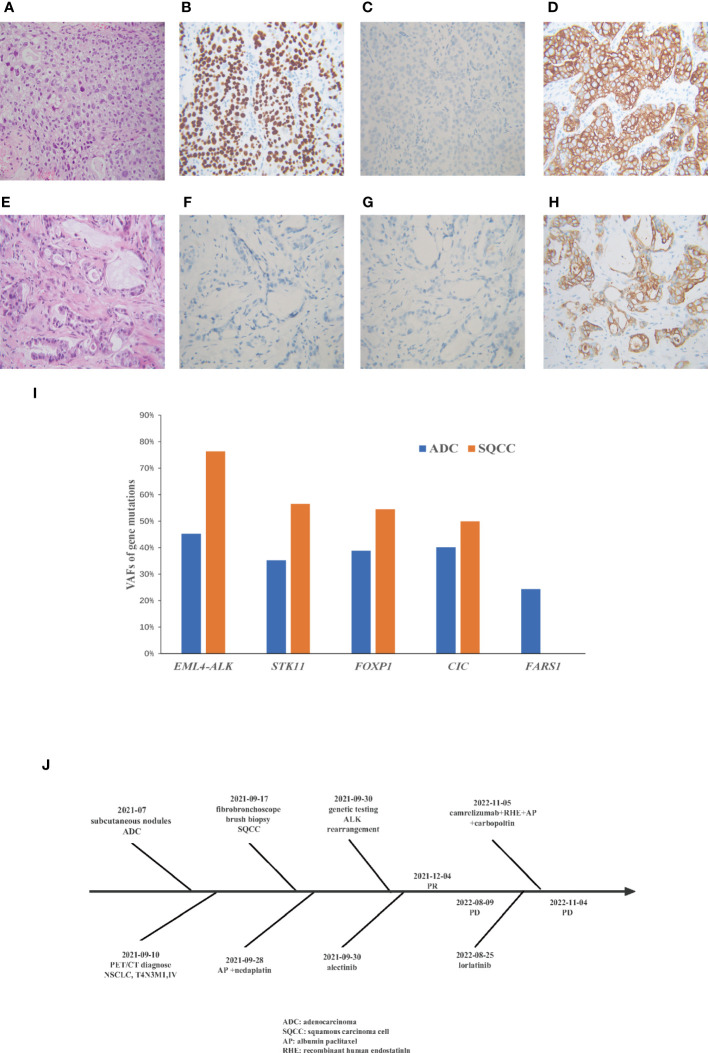
A comparison of the Hematoxylin and eosin staining results from the metastatic subcutaneous nodules and primary lung tumor. Immunohistochemical show that the lung tumor biopsy is **(A)** squamous cell cancer **(B)** positive for p40 **(C)**negative for TTF1 **(D)** positive for CK7, and metastatic subcutaneous nodules are **(E)** adenocarcinoma **(F)** negative for TTF1 **(G)** negative for Napsin A **(H)** positive for CK7. **(I)** Variant allele frequencies (VAF) of gene mutations in different morphological sites. **(J)** Flow chart of patient treatment.

A 38-year-old woman with a never smoking history, was found to have multiple head subcutaneous nodules in June 2021. The diameter of the most noticeable nodule was 0.5 cm, and the nodule was hard with an unclear boundary. In July, the nodules were found to be enlarged, and the most noticeable one had a diameter of 1 cm. Then the patient went to Zhuhai Third People’s Hospital, and she underwent a fine needle aspiration biopsy of the nodules. The pathological report revealed the nodules were adenocarcinoma (ADC). The patient felt weak and dizzy, occasionally coughed with little white sputum, and the multiple subcutaneous nodules were continually growing up. On August 10th, she got a recurrent fever for several days, and her temperature fluctuated within the range of 37.2-39°C. On September 10th, positron emission tomography/computed tomography (PET/CT) at the Fifth Affiliated Hospital of Sun Yat-sen University showed positive lesions in the left hilar central lung cancer, and multicenter peripheral lung cancer at the apical posterior end of the upper lobe of the left lung. Multiple metastases were observed in both lungs, multiple lymph nodes, bilateral pleural, the left parotid gland, bilateral mammary gland, pancreatic head, bilateral adrenal gland, bilateral kidney, bones, muscles, and subcutaneous throughout the body. On September 18^th^, the patient came to our hospital for treatment. A contrast-enhanced computed tomography (CT) scan of the chest confirmed the mass in the upper lobe of the left lung (**Figures 1A, B**). A fibrobronchoscope brush biopsy was performed on the left main bronchus tumor, and the primary provided an unexpected diagnosis of squamous cell carcinoma (SQCC) with negative results for TTF-1 and positive results for CK7 and P40 (**Figures 2A–D**). The pathological slices of the subcutaneous nodules of the scalp were borrowed from Zhuhai Third People’s Hospital. Pathological diagnosis in our hospital confirmed they were medium-differentiated ADC with a conspicuous acinar growth pattern and extracelluar mucus production (**Figure 2E**). Immunohistochemically, the nodules were negative for TTF-1, Napsin A, but showed positive expression of CK7 (**Figures 2F–H**). Given her rapidly deteriorated condition, before the NGS test report came out the patient received one cycle of chemotherapy with albumin-paclitaxel 300 mg combined with nedaplatin 80 mg injection on September 28, 2021. A panel-based next-generation sequencing (NGS) consisting of 689 cancer-associated genes (**Supplementary Table 1**) was performed individually in the primary and subcutaneous nodules. NGS indicated *ALK-EML4* (E13; A20) fusion and other mutations were shared in the two different histologic tumors with the exception of a mutation in the *FARS1* gene, which was exclusively present in subcutaneous nodules, indicating they originated from the same clone (**Supplementary Tables 2**, **3**). In the ADC of metastatic nodules, the variant allele frequency (VAF)s of *EML4-ALK*, *STK11*, *FOXP1*, *CIC*, and *FARS1* gene were 45.21%, 35.20%, 38.82%, 40.16%, and 24.35%, respectively, while in the SQCC of main lung tumor, they were 76.33%, 56.55%, 54.47%, 49.98%, and 0 correspondingly (**Figure 2I**). Finally, the patient was diagnosed with clinical stage IVB, T4N3M1c of the lung cancer possessing an *ALK* gene rearrangement and multiple distant metastases with an Eastern Cooperative Oncology Group performance status (ECOG PS) of 2-3. According to the 2021 National Comprehensive Cancer Network (NCCN) guidelines, and taking into account the patient’s ECOG PS, we interrupted the patient’s current chemotherapy regimen and started on alectinib 600 mg orally twice a day from September 30^th^. Radiotherapy was not an option due to the extensive metastasis of the tumors. Eight weeks after this administration, the efficacy was evaluated as a partial response (PR) as CT imaging showed the shrinkage of the lung (**Figures 1C, D**). Her ECOG PS decreased from 2-3 to 1, with improvement of hypoxia, cancer pain, and cachexia. Interestingly, subcutaneous nodules with ADC in morphology shrank more than the lung lesions (ADC). The efficacy of alectinib was evaluated as stable disease in May 2022. However, enlargement of subcutaneous nodules was noted in August 2022. In addition, the chest/abdomen/pelvic CT scans indicated the enlargement of the primary lesions and metastatic lesions (**Figures 1E, F**). The treatment efficacy was progressive disease (PD). Therefore, we replaced with the third-generation *ALK*-TKI lorlatinib targeted therapy on August 25^th^, 2022 for two months. The subcutaneous nodules significantly shrank and even disappeared, but the shortness of breath was aggravated after the administration of lorlatinib. She had her external plasma examined for ctDNA and found persistence of an *EML4*-*ALK* rearrangement and an extra p.E542 mutation in *PIK3CA* gene (**Supplementary Table 4**). Then the patient accepted one cycle of chemotherapy with albumin paclitaxel 350 mg and carbopoltin 400 mg combined with camrelizumab immunotherapy and recombinant human endostatinln 210 mg by injection on November 5, 2022. However, her respiratory condition deteriorated, and she died within a few days. A flowchart of the patient’s treatment timeline is provided in **Figure 2J**.

## Discussion

It is widely believed that histologic appearance is conserved when broadly metastasized. However, with other information considered, including radiographic findings and clinical features, the possibility of metastases was considered to be high in the case presented here. *EML4*-*ALK* rearrangement and other mutations shared by the two histologic sites confirmed that they are derived from a single clonal origin. It is reasonable to assume that the primary site of this case is an ASC, and the ADC component was missed due to insufficient sampling on the biopsy or the predominance of a single histology. Therefore, ASCs are known to be difficult to diagnose preoperatively (2, 6). Previously, there was one similar case, a patient was diagnosed as have SQCC and treated based on a biopsy, then performed surgical resection which revealed an ADC primary with *ALK* rearrangement (3). Compared with other similar cases, our case has some unique features. In this case, molecular profiling and variant allele frequencies of gene mutations were thoroughly assessed by a comprehensive panel of NGS in the primary and metastatic lesions, and the treatment of an ASC patient with morphological heterogeneity was comprehensively documented. The inadequacy of this study is that some of the examinations are not carried out in our hospital, therefore some figures are not provided.

ASC is relatively uncommon, accounting for 0.4-4% of all NSCLC (1). The survival for patients with ASC are statistically worse than for patients with ADC and SQCC, due to the fact that ASC patients are more likely to develop local recurrence or distant metastasis (7–9). Surgery is not advised for patients who have distant metastases (10).

Genomic was previously used to differentiate multiple primaries from metastatic lung cancer in several publications (4, 11–13). By microdissection, previous studies demonstrated ADC and SQCC of ASC shared the same genomic origin accompanying inter-component heterogeneity (5, 14). In the present case, a panel of 689 cancer-related genes was used for NGS (**Supplementary Table 1**). Our case found a high proportion of shared somatic mutations between the two tumors indicating lineage. A total of 5 somatic mutations were shared with similar VAFs (**Figure 2I**).

Our case was a female diagnosed with lung cancer at a very young age (38 years old), and she had no smoking history, which is consistent with the clinical characteristic of *ALK*-rearranged lung cancers. In addition to the *ALK* fusion, a deletion mutation in the *STK11* gene was discovered. As with tp53 mutations, co-occurring *STK11* mutations in *ALK*-rearranged NSCLC predict an unfavorable response to targeted therapy. In NSCLC, patients with *STK11* mutations had shorter overall survival (OS) and progression-free survival (PFS) than patients with *STK11* wild-type who received immunotherapy or chemotherapy (15), it also tends to suggest that this patient may have a worse prognosis. Moreover, an extra *PIK3CA* E542K oncogenic mutation was detected in the patient’s plasma after her disease progressed despite treatment with *ALK*-TKIs. Several mutations in *PIK3CA* gene has shown to result in all-generational resistance to *ALK* inhibitors (16). In addition, while *EGFR* and *ALK* mutations are more prevalent in ADC than in SQCC, *PIK3CA* mutations are more prevalent in SQCC (17). This further demonstrates that the *PIK3CA* E542K mutation, most likely from the SQCC component, was responsible for the resistance and accelerated progression of her disease during the final phases of her treatment.

Currently, there is no specific standard treatment for ASC, and current treatment of ASC is relied on the guideline for non-small cell lung cancer (NSCLC). Referring to the 2021 NCCN guidelines for NSCLC, alectinib, brigatinib, and lorlatinib are the preferable first-line *ALK*-TKIs. As the first-line therapy, alectinib showed a better efficacy than standard chemotherapy and crizotinib in *ALK*-rearranged advanced NSCLC patients (18, 19). Therefore, we decided to interrupt chemotherapy and adopted alectinib for the targeted treatment. Studies have confirmed *EGFR*-TKI significantly benefits ASC patients with *EGFR* mutations (5). However, due to the infrequent occurrence of ASC patients with *ALK* rearrangement, only a few cases reported the efficacy of *ALK*-TKIs for those patients (3, 20).

Clinically, the *ALK* rearranged SQCC patients also obtained benefit from *ALK*-TKIs, but was not as beneficial as compared to the ADC patients (21, 22). In this case, different therapeutic effects of *ALK*-TKI treatment were observed in two different morphological sites. She eventually died of lung tumor that was initially diagnosed as SQCC in a biopsy, but meanwhile her metastatic cutaneous sites almost disappeared. We speculate that ADC components are more sensitive to *ALK*-TKIs than SQCC components. Previous studies also demonstrated that TKIs had more therapeutic advantages on the ADC components than the SQCC components in treating ASC patients (20, 23).

At present, several studies have demonstrated that combining chemotherapy with *EGFR*-TKIs can prolong PFS in people with advanced *EGFR*-mutant NSCLC (24, 25). In patients with advanced *ALK*-rearranged NSCLC, clinical trials combining chemotherapy and second-generation *ALK*-TKIs are underway (26, 27). In retrospect, we might have tried the combination of chemotherapy and targeted therapy when her overall condition had improved (ECOG PS ≤ 2). We speculate that chemotherapy may be more effective in treating primary lung tumors with SQCC components which did not respond well to *ALK*-TKIs. Recent studies with small sample sizes have also looked at potential therapies for advanced ASC, such as immune checkpoint inhibitor therapy (28) and pemetrexed-based therapy (29). Further cases and investigations are needed to certify. The patient arrived at our hospital at a late stage of the disease, with extensive metastasis. Due to the fact that ASC is substantially more aggressive than ADC and SCC, early detection, diagnosis, and surgical intervention are crucial for its treatment.

## Conclusion

In conclusion, small biopsies may not accurately reflect lung cancers. In cases when these complicated tumors are only partially sampled, cautions should be advised. Molecular testing, especially a large NGS panel would not only provide key information for targeted therapy, prognosis and drug resistance, but also provide an explicit answer to confirm metastases or independent tumors. ASC patients with *ALK* rearrangement can benefit from TKI treatment, but with a different response rate in the distinctive histologic components. Further study is warranted for the diagnosis and treatment of ASCs.

## Data availability statement

The original contributions presented in the study are included in the article/**Supplementary Material**. Further inquiries can be directed to the corresponding author.

## Ethics statement

Written informed consent was obtained from the individual(s) for the publication of any potentially identifiable images or data included in this article.

## Author contributions

YY, HL, and X-sC are responsible for the conception and design of the study. Provision of patient: X-sC. Analysis of NGS data: BW, Manuscript writing: YY, HL, and X-sC. Experiment: T-hL and X-rZ. Imaging analysis: D-jZ. Final approval of manuscript: G-jZ. All authors contributed to the article and approved the submitted version.
